# Fluopsin C Promotes Biofilm Removal of XDR *Acinetobacter baumannii* and Presents an Additive Effect with Polymyxin B on Planktonic Cells

**DOI:** 10.3390/antibiotics13090875

**Published:** 2024-09-12

**Authors:** Leandro Afonso, Kathlen Giovana Grzegorczyk, Julio Martins Salomão, Kawany Roque Basso, Leonardo Cruz Alves, Maria Clara Davis Silva, Andreas Lazaros Chryssafidis, Bárbara Gionco-Cano, Sueli Fumie Yamada-Ogatta, Galdino Andrade

**Affiliations:** 1 Microbial Ecology Laboratory, State University of Londrina, Londrina 86057-970, Brazil; leandro.afonso@uel.br (L.A.);; 2 Agroveterinary Sciences Center, Santa Catarina State University, Lages 88520-000, Brazil; 3 Molecular Biology of Microorganisms Laboratory, State University of Londrina, Londrina 86057-970, Brazil; ogatta@uel.br

**Keywords:** secondary metabolite, *Pseudomonas aeruginosa* LV strain, thioformin metal complex, organometallic antimicrobial, Fluopsin C, antibiofilm effect, *Acinetobacter baumannii*

## Abstract

*Acinetobacter baumannii* emerged as one of the most important pathogens for the development of new antimicrobials due to the worldwide detection of isolates resistant to all commercial antibiotics, especially in nosocomial infections. Biofilm formation enhances *A. baumannii* survival by impairing antimicrobial action, being an important target for new antimicrobials. Fluopsin C (FlpC) is an organocupric secondary metabolite with broad-spectrum antimicrobial activity. This study aimed to evaluate the antibiofilm activity of FlpC in established biofilms of extensively drug-resistant *A. baumannii* (XDRAb) and the effects of its combination with polymyxin B (PolB) on planktonic cells. XDRAb susceptibility profiles were determined by Vitek 2 Compact, disk diffusion, and broth microdilution. FlpC and PolB interaction was assessed using the microdilution checkerboard method and time–kill kinetics. Biofilms of XDRAb characterization and removal by FlpC exposure were assessed by biomass staining with crystal violet. Confocal Laser Scanning Microscopy was used to determine the temporal removal of the biofilms using DAPI, and cell viability using live/dead staining. The minimum inhibitory concentration (MIC) of FlpC on XDRAb was 3.5 µg mL^−1^. Combining FlpC + PolB culminated in an additive effect, increasing bacterial susceptibility to both antibiotics. FlpC-treated 24 h biofilms reached a major biomass removal of 92.40 ± 3.38% (isolate 230) using 7.0 µg mL^−1^ FlpC. Biomass removal occurred significantly over time through the dispersion of the extracellular matrix and decreasing cell number and viability. This is the first report of FlpC’s activity on XDRAb and the compound showed a promissory response on planktonic and sessile cells, making it a candidate for the development of a new antimicrobial product.

## 1. Introduction

*Acinetobacter baumannii* represents one of the most threatening pathogenic bacteria worldwide [1,2]. These Gram-negative coccobacilli are strongly related to healthcare-associated infections, especially in immunocompromised patients with prolonged hospitalization [3]. In high-risk settings, such as intensive care units, *A. baumannii* has thrived, becoming endemic to these environments in many hospitals across the globe [4,5,6]. Its large repertoire of intrinsic resistance to antimicrobials, its capacity to thrive under unfavorable environmental conditions, and its ability to form biofilms are some of the factors related to the success of this pathogen in hospital settings [4,7].

Biofilm formation is an important factor regarding *A. baumannii* infections and antimicrobial resistance, since it increases its attachment and survival on multiple types of surfaces, culminating in the spread of the bacteria. Furthermore, biofilms impose a physical barrier that protects the sessile cells from inhibitory agents, such as disinfectants and antimicrobials [8,9,10]. *A. baumannii* can form biofilms on biotic and abiotic surfaces, which ultimately leads to a high rate of medical device-associated infections [11]. The most common biofilm-related infections caused by *A. baumannii* are ventilator-associated pneumonia and catheter-related infection [12]. Several factors are known to influence *A. baumannii* biofilm formation, like the proper set of biofilm-related genes of the bacteria, surface properties, and nutritional inputs. A unified model that explains the role of these determinants is still lacking [13]; however, several targets to inhibit biofilms have been determined.

Regarding antimicrobial resistance, carbapenem-resistant *A. baumannii* (CRAB) was elected by the WHO in 2017 as the top pathogen in the critical priority group for the research and development of new antibiotics [14] since only polymyxin B (PolB), colistin (polymyxin E), and tigecycline have been seen to be effective against these strains. However, polymyxin’s nephrotoxicity and rising resistance rates, in addition to tigecycline ambiguous activity, compromise clinical outcomes [15,16].

A distinctive trait of XDRAb is carbapenem resistance [5]. Data from the SENTRY Antimicrobial Surveillance Program reported an increase in the isolation of XDR *Acinetobacter calcoacetius*—*A. baumannii* complex specimens over 19 years in Latin America from 17.0% in 1997 to 86.6% in 2016 [17]. PDR isolates in Brazil accounted for 1.4% (*n* = 18) of the total 312 worldwide isolated PDR. In this alarming context, where commercial antimicrobials are no longer effective, the development of new antibiotics and antibiofilm agents derived from natural products is a necessary approach.

Fluopsin C (FlpC) (also referred to as YC 73) corresponds chemically to bis(*N*-methylthioformohydroxamato)Cu(II) and is a low-molecular-weight secondary metabolite produced by *Pseudomonas* spp. and *Streptomyces* sp. [18,19,20,21]. Its biosynthesis is triggered as a bioremediation response to high levels of intracellular cupric ions [22] in which these ions are complexed to thioformin siderophore molecules [20,23], resulting in an organometallic complex that features great antimicrobial and antitumoral activities [19,24,25,26].

This compound can be purified from microbial culture supernatants as thin dark-green-brownish prismatic crystals [18,27], and the bioprospecting of an FlpC-producing *Pseudomonas aeruginosa* LV strain in 2004 [28] enabled our group to conduct several investigations regarding its antimicrobial activity over the past 20 years. To date, we have demonstrated the efficacy of the metabolite against important human pathogens, such as KPC-producing *Klebsiella pneumoniae* strains [29], methicillin-resistant *Staphylococcus aureus* [30], and vancomycin-resistant *Enterococcus faecium* [25,31]. Nevertheless, the antibiofilm properties of the compound are still poorly investigated and therefore poorly understood.

This research aimed to evaluate the antibiofilm properties of FlpC in established biofilms of XDR clinical isolates (CIs) of *A. baumannii* as a removal agent and its interaction with PolB in planktonic cells.

## 2. Results

### 2.1. Characterization of Purified Fluopsin C

**Figure 1:**
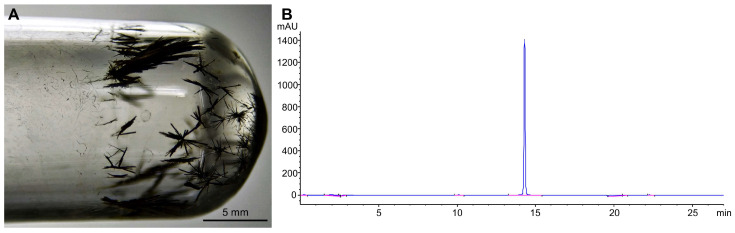
(**A**) Dark-green, thin, prismatic crystals of Fluopsin C purified from *Pseudomonas aeruginosa* LV strain supernatant by two sequential flash chromatography processes. (**B**) Purified Fluopsin C chromatogram at 262 nm using a C18 (5 µm × 4.6 mm × 250 mm) column washed with a gradient system of acidified ultrapure water (1% *v*/*v* acetic acid) to acetonitrile. High-purity (90.45%) Fluopsin C molecule was detected at 14.3 min.

Purified FlpC was morphologically characterized as dark-green, thin, prismatic crystals formed after complete solvent evaporation (Figure 1A). HPLC chromatographic analysis defined the purity of the obtained solids as 90.45% (λ = 262 nm), and FlpC presented a retention time of 14.3 min (Figure 1B). The purified and characterized compound was subsequently used in all experiments.

### 2.2. Fluopsin C Susceptibility Profiles of A. baumannii Clinical Isolates

All CIs of *A. baumannii* examined in this study were classified as extensively drug-resistant (XDR) according to Magiorakos et al.’s [32] criteria as being non-susceptible to one or more antimicrobial agents in all but one or two categories. A full description of the antimicrobial resistance profiles is presented in Table 1.

**Figure 2:**
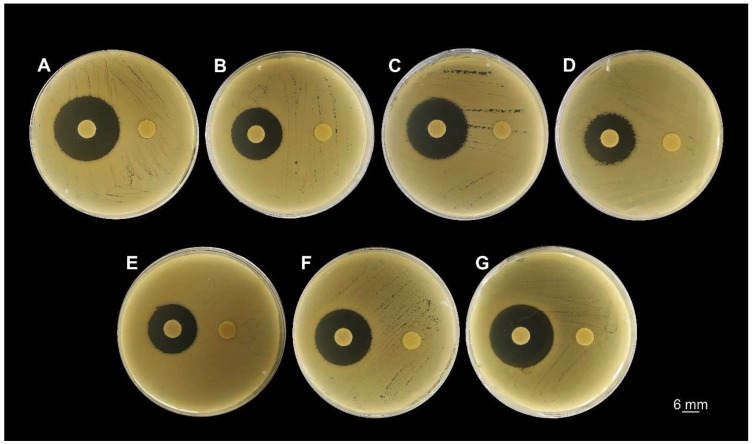
Screening for Fluopsin C susceptibility of *A. baumannii* clinical isolates and the reference strain ATCC 19606 by the disk diffusion method. The disks on the left were impregnated with 10 µg of Fluopsin C in DMSO, and the disks on the right were impregnated with 10 µL of DMSO. (**A**) ATCC 19606; (**B**) CI 223; (**C**) CI 224; (**D**) CI 226; (**E**) CI 227; (**F**) CI 230; (**G**) CI 232.

The inhibitory effect of FlpC was first screened by a disk diffusion assay and revealed the susceptibility of all strains to the natural compound (Figure 2). At 10 µg disk^−1^ concentration, FlpC promoted growth inhibition zones ranging from 16.5 ± 0.71 mm (CI 226) to 22.3 ± 0.58 mm (ATCC 19606) (Table 1). DMSO disks (control) did not promote any inhibitory effect against the tested bacteria.

Notably, MICs determined for all six CIs were equal to 3.5 µg mL^−1^ but differed from the minimal concentration needed to inhibit the growth of the reference strain ATCC 19606, which was equal to 1.75 µg mL^−1^ (Table 1).

### 2.3. Combinatory Effects of Fluopsin C with Polymyxin B

Combination assays performed with the natural antimicrobial FlpC, and the commercial antimicrobial PolB demonstrated an additive antibacterial interaction against *A. baumannii* CIs (Table 2). For this experiment, CIs were chosen to represent different ranges of MIC values for PolB. The concentration required to inhibit bacterial growth in the combination was two-fold lower for FlpC in all tests (1.75 µg mL^−1^). When combined with the natural compound, PolB dramatically decreased its MIC to a baseline of 1 µg mL^−1^ for all tested strains, indicating a fold decrease ranging from 2 to 32 (CI 223 and CI 227, respectively).

### 2.4. Growth Kinetics of Planktonic Cells Treated with Fluopsin C and Polymixin B, Alone or Combined

**Figure 3:**
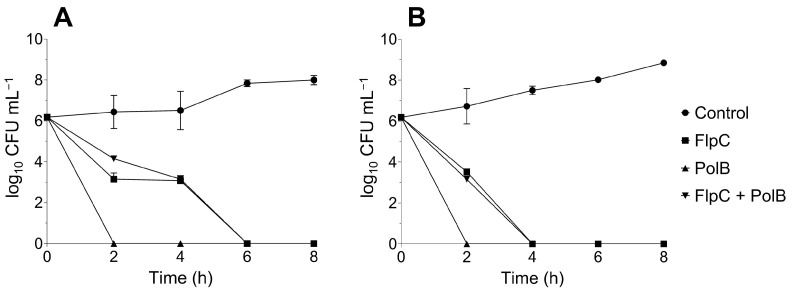
Time–kill kinetics of XDR *A. baumannii* CI 226 (**A**) and 230 (**B**) treated with MIC values to Fluopsin C (FlpC, 3.5 µg mL^−1^), polymyxin B (PolB, 226 = 8 µg mL^−1^ and 230 = 4 µg mL^−1^) and Fluopsin C/polymyxin B combination (FlpC + PolB, 1.75/1 µg mL^−1^).

The exposure of *A. baumannii* CIs 226 and 230 to FlpC and PolB, alone or combined, resulted in extensive log_10_ reductions from the first 2 h of incubation (Figure 3). PolB at the MIC values killed 100% of the cells within 2 h for both CIs. On the other hand, culture extinctions occurred after 6 and 4 h incubation for FlpC against 226 and 230 isolates, respectively. The FlpC/PolB combination resulted in a kinetic pattern of death that was similar to the one observed for cells treated with FlpC alone. The analysis was carried out for 24 h, and growth in the cultures was not observed at any subsequent time.

### 2.5. Characterization of Biofilm Formation Capacities by A. baumannii Strains

To characterize the capacity of the tested *A. baumannii* isolates to produce adherent biofilms in polystyrene surfaces, sessile cells were stained with CV after 24 h incubation at 37 °C.

The results revealed that the ATCC 19606, CI 232, and CI 224 strains are weak biofilm producers, while the CI 223, CI 226, CI 227, and CI 230 strains are moderate biofilm producers, according to the Stepanovic et al.’s [34] criteria (Table 3). None of the evaluated strains were classified as strong biofilm producers.

### 2.6. 24 h Established Biofilms on Polystyrene Removal Assay

**Figure 4:**
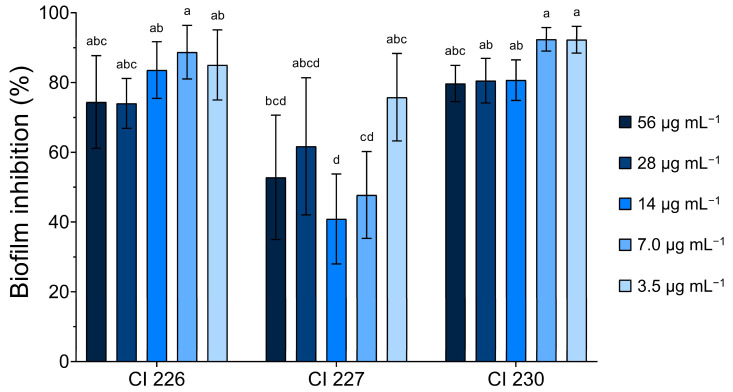
Effect of different concentrations of Fluopsin C on 24 h established biofilms of XDR *A. baumannii* clinical isolates 226, 227, and 230 (moderate biofilm producers). Different letters indicate significant differences between biofilm removal, considering *p* < 0.05 as significant in the Tukey multiple comparison test.

Three moderate biofilm-producing *A. baumannii* CIs (226, 227, and 230) were selected to examine the established biofilm removal effects of FlpC. The results revealed that the compound promoted a biofilm biomass removal activity higher than 40% at all tested concentrations (Figure 4).

One-way ANOVA indicated that the means of biofilm removal were different (*p* < 0.001) between the treatments. ATCC 19606 was used as an antibacterial-sensitive strain and weak producer of biofilms, which were completely eradicated at all tested concentrations.

CI 226 and CI 230 removal percentages presented no significant differences when compared. In contrast, CI 227 exhibited more variable results towards FlpC action on its biofilms, and the lowest reduction (40.88 ± 12.89%) was observed with the compound at 14 µg mL^−1^ (4 × MIC). For the other isolates, a percentage of biofilm removal greater than 70% was observed at all FlpC concentrations.

The higher percentage removal of biofilms occurred at 7.0 µg mL^−1^ for CI 226 (88.72 ± 7.69%) and 230 (92.40 ± 3.38%), and at 3.5 µg mL^−1^ for CI 227 (75.79 ± 12.54%). No significant differences were found when comparing MIC or 2 × MIC values in terms of performance on biofilm removal, which ultimately led to the use of the MIC of 3.5 µg mL^−1^ in further assays. These results support that low concentrations of FlpC can remove the majority of 24 h formed biofilms of tested bacteria.

### 2.7. Microscopic Analyses of 24 h Biofilm Removal at MIC on a Glass Surface

**Figure 5:**
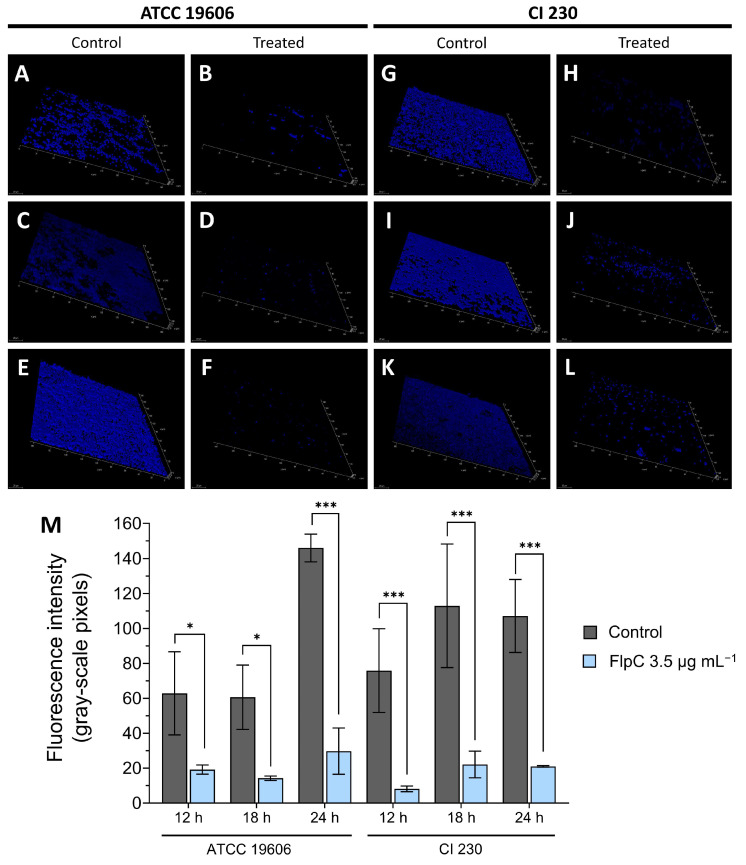
Temporal effect of exposure of *A. baumannii* ATCC 19606 and XDR CI 230 24 h biofilms to Fluopsin C at MIC for planktonic cells (3.5 µg mL^−1^). Fluorescence microscopy tridimensional reconstruction of ATCC 19606 24 h biofilms treated for 12 h (**A**,**B**), 18 h (**C**,**D**), and for 24 h (**E**,**F**), and CI 230 24 h biofilms treated for 12 h (**G**,**H**), 18 h (**I**,**J**), and 24 h (**K**,**L**). Controls consisted of the addition of TSB-G without Fluopsin C (**A**,**C**,**E** for ATCC 19606 and **G**,**I**,**K** for CI 230). Treatments were based on the addition of TSB-G plus 3.5 µg mL^−1^ and incubation at 37 °C during different times (**B**,**D**,**F** for ATCC 19606 and **H**,**J**,**L** for CI 230). (**M**) Comparison of fluorescence intensity (gray-scale pixels) of ATCC 19606 and CI 230 24 h biofilms treated over 12, 18, or 24 h with 3.5 µg mL^−1^ FlpC. Asterisks represent significative differences between control and treatment by Sidak’s multiple-comparisons test where *p* ≤ 0.05 (*); *p* ≤ 0.001 (***).

A microscopic analysis of 24 h biofilm exposed to 3.5 µg mL^−1^ of FlpC revealed a significant removal of sessile cells from the glass surface over time (Figure 5) (one-way ANOVA, *p* < 0.0001). When compared to the untreated controls, all treatments resulted in an inhibition higher than 60%. A percentage reduction in the fluorescence intensity of ATCC 19606 sessile cells was observed: 69.48% (*p* = 0.0259), 76.48% (*p* = 0.0163), and 79.62% (*p* < 0.0001) after 12, 18, and 24 h of incubation, respectively. In contrast, CI 230 displayed 89.24% of the established biofilm removed after 12 h (*p* = 0.0003) of exposure, 80.40% after 18 h (*p* < 0.0001), and 80.49% after 24 h (*p* < 0.0001). We believe that the remaining fluorescence was due to DAPI binding to cellular residues and did not represent viable cells.

### 2.8. Live/Dead Stain of Fluopsin C-Treated Biofilm

**Figure 6:**
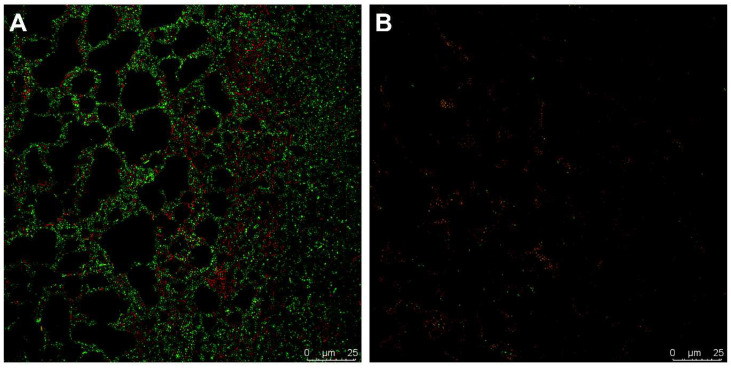
Live/dead staining micrography of 24 h biofilms of XDR *A. baumannii* CI 230 treated with Fluopsin C. (**A**) Untreated biofilm controls; (**B**) treatment with 3.5 µg mL^−1^ Fluopsin C for 12 h.

The live/dead staining of a 24 h biofilm of CI 230 revealed that after 12 h of treatment with FlpC (3.5 µg mL^−1^), the biofilm was completely disrupted and the majority of the remaining cells were not viable (Figure 6). No adhered cells were observed after FlpC exposure for prolonged periods (18 and 24 h).

## 3. Discussion

Therapeutical options are limited for CRAB treatment and monotherapy seems to be no longer effective, jeopardizing clinical outcomes and elevating health costs. Therefore, the development of new antibiotics is necessary [1,14]. Previous studies carried out by our group indicated the in vitro efficiency of FlpC, a secondary metabolite produced by the *P. aeruginosa* LV strain, against human and plant pathogens, making it a promising candidate for new drug development.

In this study, we first investigated the efficacy of FlpC against XDRAb planktonic cells. FlpC was obtained from cultures of *P. aeruginosa* LV strain under copper stress. The organocupric molecule was purified from the cell-free supernatant through novel flash chromatography methods until it reached 90.45% purity.

In the initial screening using the disk diffusion assay, all the *A. baumannii* CIs tested presented inhibition zones greater than 16 mm for FlpC 10 µg disks. The MIC of FlpC in planktonic cells was equal to 3.5 µg mL^−1^ for all isolates and 1.75 µg mL^−1^ for ATCC 19606. Other *A. baumannii* strains had their FlpC MIC characterized by Patteson et al. [35], revealing different patterns of *A. baumannii* susceptibility to FlpC, ranging from 5.2 µg mL^−1^ (ATCC 19606) to 20.8 µg mL^−1^ (AR0312 strain). Currently, FlpC produced by the *P. aeruginosa* LV strain and PAO1 presents different hypotheses regarding its biosynthesis pathways [36]. It is still unclear how FlpC is produced by different bacteria and if there are any minor differences between the final product derived from different *Pseudomonas* species. Other ESKAPEE pathogens (*E. faecium*, *S. aureus*, *K. pneumoniae*, *A. baumannii*, *P. aeruginosa*, *Enterobacter* spp., and *Escherichia coli*) are also known to be susceptible to FlpC, such as KPC-producing *K. pneumoniae* kpn-19 (MIC = 2.0 µg mL^−1^), methicillin-resistant *S. aureus* N315 (MIC = 0.5 µg mL^−1^), *S. aureus* BEC9393 (MIC = 1.0 µg mL^−1^), and vancomycin-resistant *E. faecium* VRE-170 (MIC = 1.0 µg mL^−1^) [25,31].

The last update of the Infectious Diseases Society of America (IDSA) guidelines recommends the use of polymyxins, cefiderocol, and high doses of minocycline, tigecycline or ampicillin–sulbactam (a saturation of sulbactam) for CRAB treatment, all used in combination therapy regimens with at least one more antimicrobial agent [37]. CLSI also recommends that polymyxins should be used with one or more antimicrobial agent [38]. Considering the approach of combination therapy, we combined FlpC and PolB to determine their interactional effects, which resulted in additive antibacterial activity. Decreases of 2-fold to 32-fold were observed in the MIC values of PolB in XDRAb in the present study. When combined, the concentration required for FlpC and PolB effectiveness reaches a baseline of 1.75 and 1 µg mL^−1^, respectively, for all isolates.

The time–kill assay revealed that cells of XDRAb were killed after 4 to 6 h of incubation in the presence of FlpC 3.5 µg mL^−1^ or 1.75/1 µg mL^−1^ FlpC/PolB. We highlight that the additive effect between the compounds may rely on tackling adaptive resistance to PolB [39], and this must be investigated in further research from our group.

Polymyxin action relies on its interaction with negatively charged lipid A from LPS on the outer membrane, triggering the displacement of Ca^2+^ and Mg^2+^ that are crucial for membrane stabilization, leading to the uptake of the antibiotic into the periplasmic space and membrane breakage [40]. The major *A. baumannii* resistance mechanisms to polymyxins occur by alterations in LPS or a complete lack of this structure [41]. FlpC also acts on the cytoplasmatic membrane of bacteria, causing damage to its structure and ultimately resulting in increased permeability and cell lysis [31]. Activity affecting common targets by the antimicrobials used in this study may explain the additive effect observed. Combinatory regimens with antimicrobials that act on different bacterial structures (or processes) to the membranes may provide differential interactions with FlpC, lowering MIC values required for its antibacterial performance.

Natural products (NPs) constitute a promising source for new antimicrobial development while also being considered environmentally friendly and self-sustaining, usually related to low resistance frequencies [42]. However, limited reported data have shown the antibiofilm properties of microbial metabolites affecting *A. baumannii* strains. On this subject, the most studied NPs are phytochemicals [43,44,45,46,47]. Little is known about FlpC effects on these bacterial communities. 

To the best of our knowledge, this is the first report of an assessment on the antibiofilm properties of FlpC in XDRAb. Previous studies of our group indicated that semi-purified fractions of *P. aeruginosa* LV cultured under copper stress presented inhibitory effects on biofilms. Cultures of the phytopathogen *Xanthomonas citri* pv. citri 306 exposed to 200 µg mL^−1^ F3 had their exopolysaccharides’ biofilm content removed along with morphological alterations to the cells with no lysis after 1 h of exposure. The effect evolved to cell lysis within 6 h [48]. The FlpC content in F3 was not determined.

Similarly, Lopes et al. [49] reported that *Xanthomonas axonopodis* BSC475a cultures treated with 200 µg mL^−1^ F3 for 1 h presented an absence of EPS and cell wall alterations. After 3 h, cell lysis was frequently observed in the cultures. *X. citri* pv. citri 306 produces a thick layer of EPS on orange leaves infected with the bacteria. After the treatment of leaf surfaces with curative or preventive regimens consisting of the application of F3d 10 µg mL^−1^, a reduction in EPS and alterations in the cell ultrastructure were observed [50].

Regarding ESKAPEE pathogens, 62.5 µg mL^−1^ of the F3d fraction inhibited 24 h biofilms of KPC-producing *K. pneumoniae* KPC-3, decreasing the number and viability of sessile cells, as well as the presence of extracellular material, in addition to prominent morphological changes after 24 h of exposure [29]. F3d is the result of a purification method of F3 by vacuum liquid chromatography, and FlpC represents 30% of F3d content [50]. Established biofilms (24 h) of 18 methicillin-resistant *Staphylococcus aureus* (MRSA) had their metabolic activity affected by exposure to different F4A concentrations [51]. In that study, 6.25 µg mL^−1^ was the necessary concentration to inhibit 50% of the metabolic activity of the biofilm (bMIC_50_) in most MRSA isolates (*n* = 12). For a 90% inhibition (bMIC_90_), the value shifted to 12.5 µg mL^−1^ (*n* = 10). The remaining isolates presented bMIC_90_ ≥ 25 µg mL^−1^ (*n* = 8). FlpC content in F4A is about 25% [21].

Recently, the effect of F4A on biofilms of *Candida auris* was also investigated [26]. F4A was able to reduce 50% of CFU counts of sessile cells (SMIC_50_) at concentrations of 32.94 µg mL^−1^ in *C. auris* CBS 10913 and 47.02 µg mL^−1^ in CBS 12766. The inhibition of 80% of CFU counts (SMIC_80_) required 100 µg mL^−1^ for both strains. Another finding of the study was the synergistic interaction of F4A with biologically synthesized silver nanoparticles (bioAgNP). SMIC_50_ in a combination regimen achieved values of F4A/bioAgNP with a proportion of 12.40/13.21 and 9.26/10.21 µg mL^−1^ for CBS 10913 and CBS 12766, respectively.

It seems that the antibiofilm activity of FlpC and FlpC-containing fractions follows a pattern in their action. First, the extracellular components of the biofilm community are removed [48]. Then, the bacterial cells die in a time-dependent manner due to the accumulation of ultrastructural alterations, as would occur when they are exposed to the compound in planktonic state. In this sense, it is reasonable to suggest that the antibiofilm properties of FlpC on bacterial biofilms may be linked to EPS dispersion, thereby guiding the cells within the biofilm to become exposed to the compound, culminating in cell lysis and eventually biofilm elimination.

We believe that this would demand FlpC to be stable enough to act in the EPS dispersion and against the bacterial cells without being consumed, degraded, or converted into other molecules. The nature of EPS dispersion caused by FlpC and its stability while performing effector mechanisms within a multi-faceted living environment still demands further investigations.

Another interesting finding is that the MIC of FlpC in planktonic cells of XDRAb (3.5 µg mL^−1^) retained a strong biomass removal capacity on established biofilms, surpassing 70% removal in most treatments. It is known that a biofilm may be more resistant to antibiotic action, requiring concentrations 10–1000 fold higher when compared to planktonic cells [52,53]. Therefore, the maintenance of a low concentration necessary for the strong biomass removal of the bacterial community reinforces the potential of FlpC as an antibiofilm agent.

Studies with FlpC have suggested that this secondary metabolite is a great candidate for new antimicrobial drug development, and its effectiveness against a large repertoire of human and plant pathogens is being demonstrated. In the future, investigations regarding the compound should focus on determining the specific mechanisms of inhibition over biofilms, the proposition of a methodology for its use as an antimicrobial/antibiofilm agent, and the characterization of possible toxic effects associated with its use for safety assessments.

## 4. Materials and Methods

### 4.1. Microorganisms

Six XDRAb isolates were provided from the collection of microorganisms of the Clinical Microbiology Laboratory of the State University of Londrina (Londrina, Paraná, Brazil). All isolates were recovered from tracheal secretions and were selected according to their antibacterial resistance profiles. XDRAb and the reference strain *A. baumannii* ATCC^®^ 19606™ were stored in tryptic soy broth (TSB) (Merck, Darmstadt, Germany) plus 40% (*v*/*v*) glycerol at −20 °C.

The FlpC-producing *P. aeruginosa* LV strain (GenBank nº CP058323, BioProject PRJNA450135, BioSample SAMN08930812) was isolated from old leaf lesions of an orange tree (*Citrus sinensis* cv. Valencia) with citrus canker disease caused by *Xanthomonas axonopodis* pv. citri at Astorga city, Paraná, Brazil [28]. The LV strain was maintained on nutrient broth (NB) plus 0.1 g L^−1^ CuCl_2_∙2H_2_O and 40% (*v*/*v*) glycerol at −20 °C.

### 4.2. Antimicrobial Susceptibility Testing of Clinical Isolates

Antimicrobial susceptibility profiles of *A. baumannii* clinical isolates (CIs) were determined by the Vitek^®^ 2 Compact (bioMérieux, Marcy-l’Etoile, France) automated system for the following antimicrobials: amikacin, gentamicin, ampicillin-sulbactam, ceftazidime, ceftriaxone, cefepime, imipenem, meropenem, piperacillin-tazobactam, ciprofloxacin, and colistin.

Disk diffusion assays were performed for amikacin, gentamicin, levofloxacin, trimethoprim-sulfamethoxazole, and tetracycline. Susceptibility to PolB and tigecycline was evaluated by the broth microdilution method. The results were interpreted according to the Clinical and Laboratory Standards Institute cut-off values [33], and based on the resistance profiles, the clinical isolates were classified as proposed by Magiorakos et al. [32].

### 4.3. Fluopsin C Production and Extraction from the Supernatant

The FlpC production process followed the patented procedure PI0803350-1 [54] with the modifications proposed by Bedoya et al. [55]. From stock cultures, the *P. aeruginosa* LV strain was cultivated twice in nutrient agar plus copper chloride (g L^−1^), peptone 5.0; meat extract 3.0; copper chloride (CuCl_2_∙2H_2_O) 0.1; and agar 15.0 (pH 7.0 ± 0.2), at 28 °C for 48 h and 24 h, respectively. 

Bacterial colonies were resuspended in sterile 0.85% (*w*/*v*) NaCl solution and the cell density was adjusted to an OD_590nm_ of 0.09 [10^8^ colony-forming units (CFU) mL^−1^]. Erlenmeyer flasks (500 mL capacity) containing 100 mL of NB plus copper chloride (g L^−1^): peptone 2.0; meat extract 1.2; and CuCl_2_∙2H_2_O 0.03 (pH 6.8 ± 0.2), were inoculated with 0.01% (*v*/*v*) of the adjusted cellular suspension and incubated for 8 days at 28 °C and 170 rpm in an orbital shaker (Lab Companion IS-971, Jeio Tech, Seoul, Republic of Korea).

LV cultures were centrifuged at 9000 rpm and 4 °C for 15 min, and the cell-free supernatants were acidified to pH 4.0 with an HCl (1 M) solution and further centrifugated at the same conditions. The acidified cell-free supernatant was dried for 48 h at 60 °C. Dried extracts were submitted to liquid–liquid extraction (three times) using dichloromethane at 1:2 (*v*/*v*). The dichloromethane phase (DP) was collected and concentrated using the rotary evaporator Rotavapor R-215 (Büchi, Flawil, Switzerland).

### 4.4. Fluopsin C Purification by Flash Chromatography

FlpC was purified from the DP through two sequential procedures of flash chromatography (FC), both using 10 mm diameter glass columns filled with 15 cm of a silica gel 60 (0.040–0.063 mm/230–400 mesh ASTM) (Macherey-Nagel, Düren, Germany) slurry packed with n-hexane. In the first FC, the mobile phase was composed of (*v*/*v*) dichloromethane/ethyl acetate (95:5), and in the second, FC dichloromethane/ethyl ether (90:10).

Fractions of approximately 1 mL were collected and submitted to thin-layer chromatography (TLC) performed on Alugram Xtra SIL G/UV_254_ pre-coated sheets (0.20 mm silica gel 60 layer) (Macherey-Nagel, Düren, Germany) using a mixture of (*v*/*v*) cyclohexane/chloroform/methanol (45:45:10) as the mobile phase. Chromatogram spots were observed under UV 254 and 366 nm. The purest fractions of the compound were combined, characterized by high-performance liquid chromatography (HPLC), and used as the standard antimicrobial.

### 4.5. Fluopsin C Characterization by HPLC

Purified FlpC was characterized by HPLC using Agilent 1260 Infinity (Agilent ChemStation software v. C01.05, Agilent Technologies, Santa Clara, CA, USA) with an adapted method from Bedoya et al. [55]. The analysis was performed using the C18 reverse-phase column Zorbax 300SB-C18 (5 µm × 4.6 mm × 250 mm) (Agilent Technologies, USA) and a gradient system of acidified ultrapure water (1% *v*/*v* of acetic acid) (A) to acetonitrile (B) at a flow rate of 1 mL min^−1^ under the following conditions (%): (A) 99:01 (B) to (A) 85:15 (B) for 2 min; (A) 17:83 (B) for 20 min; and returning to (A) 99:01 (B) for 2 min and staying in this condition for 3 min (total run time = 27 min).

FlpC samples were prepared by diluting 1 mg of the compound in 1 mL of acetonitrile, and the solution was filtered through a 0.22 µm membrane (20 mm diameter filter) (Macherey-Nagel, Düren, Germany). Moreover, 10 µL of the sample was injected into the equipment, and the presence of FlpC was monitored at a 262 nm wavelength and at an averaged retention time of 14 min.

### 4.6. Determination of Acinetobacter baumannii Fluopsin C Susceptibility by Agar Diffusion Method

The disk diffusion assay was performed according to CLSI guidelines [56]. Paper disks (6 mm) were impregnated with FlpC diluted in 100% dimethyl sulfoxide (DMSO) at a concentration of 10 µg disk^−1^. Negative controls consisted of a 10 µL disk^−1^ of DMSO. The plates were incubated for 24 h at 37 °C, and the antimicrobial activity of the compound was evaluated by measuring the inhibition zone diameters (mm) formed around the disks. The test was performed twice with independent XDRAb cultures and the results were averaged.

### 4.7. Determination of the Minimum Inhibitory Concentrations (MICs)

MICs of FlpC against *A. baumannii* CIs and ATCC 19606 were determined by following CLSI guidelines for the broth microdilution method [57] with adaptations. The tests were performed in sterile flat-bottomed polystyrene 96-well plates (Sorfa Life Science Research Co., Huzhou, China). FlpC was first diluted into 100% DMSO, followed by two-fold dilutions to achieve test concentrations ranging from 56 to 0.11 µg mL^−1^ in cation-adjusted Mueller–Hinton broth (CAMHB) (Neogen, Lansing, MI, USA). The final concentrations of DMSO incorporated into the dilution were between 1.12% and 0.002% (*v*/*v*). CAMHB alone and CAMHB inoculated with bacteria were considered the sterility and positive growth controls, respectively.

After 20 h of incubation at 37 °C, 20 µL of 1% (*w*/*v*) 2,3,5-triphenyl-2H-tetrazolium chloride (TTC) (Sigma-Aldrich, Saint Louis, MO, USA) was added to each well following an additional incubation for 30 min at 37 °C. This method is based on a reduction in colorless TTC by metabolic active cells, resulting in the formation of red-colored formazan. MICs were determined as the lowest concentrations of FlpC that completely inhibited the visual metabolic activity of the tested bacteria. The test was performed twice in triplicate on different occasions.

### 4.8. Interaction Assay by the Microdilution Checkerboard Method

To evaluate the effects of FlpC combined with PolB, the checkerboard method was performed. Both antimicrobials were first diluted in CAMHB. The FlpC solution was two-fold diluted to concentrations ranging from 14 to 0.109 µg mL^−1^ directly in 96-well plates. PolB sulfate 500.000 IU (Eurofarma, São Paulo, Brazil) was diluted two-fold separately to achieve concentrations ranging from 32 to 0.5 µg mL^−1^, and then it was transferred to the respective wells.

The inoculum for the test was based on a final cellular density of 10^5^ CFU mL^−1^ per well and was prepared identically to the inoculum for MICs. CAMHB inoculated with bacteria and CAMHB alone were considered the growth control and sterility control, respectively. Plates were incubated for 20 h at 37 °C, and TTC was used to assess the metabolic activity of the bacterial cells.

The fractional inhibitory concentration index (FICI) was calculated and interpreted, as recommended by Sopirala et al. [58], as represented in Equation 1, being the sum of the fractional inhibitory concentrations (FICs) of FlpC and PolB (Equations (2) and (3), respectively).

(1) $$FICI={FIC}_{fluopsin\;C}+{FIC}_{polymyxin\;B}$$

(2) $${FIC}_{fluopsin\;C}=\left(\frac{MIC\;of\;fluopsin\;C\;in\;combination}{MIC\;of\;fluopsin\;C\;alone}\right)$$

(3) $${FIC}_{polymyxin\;B}=\left(\frac{MIC\;of\;polymyxin\;B\;in\;combination}{MIC\;of\;polymyxin\;B\;alone}\right)$$

The interactions of FlpC with PolB were interpreted according to the FICI values, being categorized as synergism when FICI ≤ 0.5; additivity when 0.5 < FICI ≤ 1.0; no interaction when 1.0 < FICI ≤ 4.0; and antagonism when FICI > 4.0. Tests were performed twice using independent cultures.

### 4.9. Time–Kill Kinetics

The temporal inhibition of XDRAb 226 and 230 by FlpC and PolB, alone or combined, was determined by a time–kill assay. The experiment was performed in flat-bottom polystyrene 12-well plates containing 2 mL of CAMHB supplemented with the respective MIC values for FlpC or PolB and the FICI value for the combination. Bacterial inocula consisted of 1.5 × 10^6^ CFU mL^−1^ and the treatments were incubated for 24 h at 37 °C. CAMHB without the addition of any antimicrobial was used as the control. Aliquots of 100 µL were collected every 2 h and serially diluted (1:10) in 0.85% NaCl (saline), and 10 µL was plated onto MHA (via the drop plate method). CFU counting was performed after 24 h of incubation at 37 °C, and the averaged data were expressed as log_10_ CFU mL^−1^. The experiment was performed in triplicate on two independent occasions.

### 4.10. Biofilm Formation and Quantification Assay by the Crystal Violet Method

Biofilm formation capacity by *A. baumannii* CIs was examined through the adapted crystal violet (CV) method from Stepanović et al. [34]. Briefly, 24 h growth colonies in MHA were resuspended in 5 mL of TSB plus 1% (*w*/*v*) glucose (TSB-G) and incubated for 18 h at 37 °C. The cell density of the cultures was adjusted to 0.5 McFarland and then diluted 1:100 in the same broth (~10^6^ CFU mL^−1^). Moreover, 200 µL of these cellular suspensions were added to sterile flat-bottomed polystyrene 96-well plates (Sorfa Life Science Research Co., Huzhou, China) and incubated aerobically for 24 h at 37 °C. Negative controls consisted of TSB-G-filled wells and, to avoid edge effects, the extremities of the wells were filled with sterile water [59].

Afterwards, the wells had their broth aspired and were washed three times with sterile saline. Biofilms were allowed to dry at room temperature for 15 min and then were fixed with 200 µL of 100% methanol at the same time. After methanol removal, the plates were dried, as previously mentioned, and biofilms were stained with a 0.1% (*w*/*v*) aqueous CV solution for 15 min. The excess stain was removed by three washings with sterile saline solution, and the wells were dried once more under the same conditions. Finally, the CV was resolubilized with 200 µL of methanol and the OD was measured using an Asys UVM 340 scanning microplate reader (Biochrom, Cambridge, UK) at λ = 570 nm. The tests were performed in quintuplicate with three independent repetitions.

Cut-off values (ODc) were established according to Stepanović et al. [34] and were expressed as the sum of the averaged OD_570nm_ of the negative control with three times its standard deviation (SD) (Equation (4)).

(4) $$ODc=averaged\;{O.D.}_{570nm}+\left(3\times SD\right)$$

Based on the amount of adherent biofilm formed in 24 h, the strains were classified as having no biofilm producer when OD ≤ ODc; a weak biofilm producer when ODc < OD ≤ 2 × ODc; a moderate biofilm producer when 2 × ODc < OD ≤ 4 × ODc; and a strong biofilm producer when OD > 4 × ODc.

### 4.11. Removal of Established Biofilm

The capacity of FlpC to remove established biofilms was examined as described by Bardbari et al. [9], with modifications. In these experiments, *A. baumannii* CIs and the reference strain were allowed to form biofilms as described before. The media were aspired and the wells were washed three times with 200 µL of sterile saline, followed by drying at room temperature for 15 min.

FlpC was serially two-fold diluted in TSB-G, achieving concentrations ranging from 56 to 3.5 µg mL^−1^, and 200 µL of the antimicrobial-containing broth was added to the wells. Treated biofilm plates were aerobically incubated for 24 h at 37 °C. For all assays, FlpC-free wells (untreated) and biofilm-free wells (negative control [nc]) were included as controls. After the incubation, biofilms were processed and stained as described previously. The percentage of biofilm removed after exposure to different concentrations of FlpC was calculated according to Pitts et al. [60] (Equation (5). The tests were performed three times with independent cultures and the results were averaged.

(5) $$Biofilm\;removal\left(\%\right)=\left(\frac{\left(ODuntreated-ODnc\right)-\left(ODtreatment-ODnc\right)}{\left(ODuntreated-ODnc\right)}\right)*100$$

### 4.12. Temporal Dynamics of Fluopsin C Removal Activity Using Confocal Laser Scanning Microscopy (CLSM) on 24 h Biofilms Formed on a Glass Surface

The temporal effects of FlpC on the biofilms of *A. baumannii* ATCC 19606 and CI 230 were examined using DAPI (4′,6-diamidino-2-phenylindole) staining. Moreover, 2 mL of bacterial suspensions in TSB-G (10^6^ CFU mL^−1^) were added to sterile flat-bottomed polystyrene 12-well plates (Kasvi, Pinhais, Brazil), and sterile squared glass coverslips (22 × 22 mm) treated with a 1% (*v*/*v*) acetic acid solution were aseptically positioned inside the wells. The plates were aerobically incubated for 24 h at 37 °C.

After the incubation, biofilms were formed both in the wells and on the glass coverslips. Planktonic cells were removed by triple washing with sterile saline solution, and then 2 mL of TSB-G containing 3.5 µg mL^−1^ of FlpC (2 × MIC for ATCC 19606 and MIC for CI 230) was added to the wells. After treatment, the plates were subjected to additional incubation. Controls were based on the addition of TSB-G without FlpC.

After 12, 18, and 24 h of exposure, the glass coverslips were washed, dried, and fixed with paraformaldehyde 4% (*v*/*v*) for 20 min at 37 °C. Biofilms were stained with DAPI (5 µg mL^−1^ in phosphate-buffered saline [PBS]) for 20 min at 37 °C, then were subsequently washed three times with PBS and observed under a Leica SP8 confocal microscope (Leica Microsystems, Wetzlar, Germany) using excitation/emission wavelengths of 405 nm/415–485 nm. A series of three fields per coverslip were analyzed regarding fluorescence intensity.

### 4.13. Live/Dead Stain of CI 230 24 h Biofilm Treated with Fluopsin C

The viability of sessile cells of CI 230 biofilms was examined using BacLight LIVE/DEAD bacterial viability kit L7012 (Invitrogen, Waltham, MA, USA) staining. Glass coverslips with biofilms were prepared and treated a described in Section 4.12.

After washing the coverslips with sterile saline, the coverslips were dried at room temperature for 10 min. Sessile cells were stained with a 1:1 (*v*/*v*) mixture of SYTO 9 (3.34 mM) and propidium iodide (20 mM) in sterile saline for 15 min in the dark. After that, biofilms were observed under a Leica SP8 confocal microscope (Leica Microsystems, Germany) using excitation/emission wavelengths of 480/500 nm (SYTO 9) and 490/635 nm (propidium iodide).

### 4.14. Statistical Analysis

The results are averaged and are presented with the standard deviation. Biofilm removal assays were analyzed through one-way ANOVA and Tukey’s multiple comparison test to investigate differences in all the treatments regarding FlpC performance in biofilm removal. Fluorescence intensity data were analyzed by one-way ANOVA followed by Sidak’s multiple comparison of each treatment with its specific control. All analyses were performed using GraphPad Prism 8.0 (GraphPad Software, Boston, MA, USA) considering *p* < 0.05 as indicative of a significant difference.

## 5. Conclusions

The present study reports for the first time the use of FlpC against XDRAb planktonic cells and biofilms. FlpC was effective against planktonic cells of XDRAb, alone or in combination with PolB. FlpC acts over 24 h established biofilms of XDRAb, promoting the depletion of biofilm extracellular components and decreasing cell number and viability at low concentrations. Based on the experimental data and prior studies by our group, FlpC is a promising candidate for new drug development due to its broad inhibitory effects. Future studies should explore the cytotoxic effects of the compound, investigate delivery systems capable of attenuating its deleterious effects, and indicate potential usage as an antimicrobial and antibiofilm product along with its safety assessments.

## Figures and Tables

**Table 1:** Antimicrobial susceptibility profiles of *Acinetobacter baumannii* for commercially available antimicrobials and Fluopsin C. The antimicrobial effect of Fluopsin C was determined by measuring the inhibition zones (mm) obtained by the disk diffusion method (10 µg disk^−1^) and MICs (µg mL^−1^) were determined by the broth microdilution method.

*A. baumannii*	Phenotypic Resistance Profile ^1^	Inhibition Zone FlpC (mm)	MIC FlpC (µg mL^−1^)
ATCC^®^ 19606™	-	22.3 ± 0.58	1.75
CI 223	AMK, GEN, IPM, MEM, CIP, LVX, CAZ, CRO, FEP, SXT, SAM, CST	18.5 ± 0.71	3.5
CI 224	AMK, GEN, IPM, MEM, CIP, LVX, CAZ, CRO, FEP, SXT, SAM, CST, PolB	18.0 ± 1.41	3.5
CI 226	AMK, IPM, MEM, CIP, TZP, CAZ, CRO, SXT, PolB	16.5 ± 0.71	3.5
CI 227	AMK, IPM, MEM, CIP, LVX, CAZ, CRO, FEP, SXT, SAM, CST, PolB	18.5 ± 0.71	3.5
CI 230	AMK, GEN, IPM, MEM, CIP, LVX, CAZ, CRO, FEP, SXT, SAM, PolB, TET	17.3 ± 1.53	3.5
CI 232	AMK, GEN, IPM, MEM, CIP, LVX, CAZ, CRO, FEP, SXT, SAM, PolB	20.0 ± 1.41	3.5

^1^ Amikacin = AMK; gentamicin = GEN; imipenem = IPM; meropenem = MEM; ciprofloxacin = CIP; levofloxacin = LVX; piperacillin-tazobactam = TZP; ceftriaxone = CRO; ceftazidime = CAZ; cefepime = FEP; trimethoprim-sulphamethoxazole = SXT; ampicillin-sulbactam = SAM; colistin = CST; polymyxin B = PolB; tetracycline = TET.

**Table 2:** Effect of Fluopsin C in combination with polymyxin B on planktonic cells of XDR *A. baumannii* clinical isolates. Polymyxin B susceptibility profiles are represented as resistant (R) and intermediate (I), according to CLSI’s M100 standards [33].

Strain	MIC FlpC (µg mL^−1^)		MIC PolB (µg mL^−1^)		FICI	Interaction Type
	Alone	Combined	Alone	Combined		
CI 223	3.5	1.75	2 (I)	1 (I)	1.00	Additive
CI 226	3.5	1.75	8 (R)	1 (I)	0.62	Additive
CI 227	3.5	1.75	32 (R)	1 (I)	0.53	Additive
CI 230	3.5	1.75	4 (R)	1 (I)	0.75	Additive

**Table 3:** Characterization of biofilm production by XDR *A. baumannii* clinical isolates and the reference strain ATCC 19606 on a polystyrene surface. Biofilms were produced over 24 h at 37 °C in sterile flat-bottomed 96-well polystyrene plates and stained with 0.1% (*w*/*v*) crystal violet aqueous solution. The stain was resolubilized in methanol and absorbance was measured at 570 nm wavelength.

ID	OD_570nm_	Biofilm Production
Control	0.160 ± 0.020	-
CI 223	0.579 ± 0.033	Moderate
CI 224	0.294 ± 0.083	Weak
CI 226	0.557 ± 0.079	Moderate
CI 227	0.690 ± 0.063	Moderate
CI 230	0.605 ± 0.074	Moderate
CI 232	0.231 ± 0.025	Weak
ATCC 19606	0.201 ± 0.016	Weak

## Data Availability

The original contributions presented in the study are included in the article, further inquiries can be directed to the corresponding author.
